# Valorization of Sea Buckthorn, Black Chokeberry, and Black Currant Branch Biomass as a Novel Source of Bioactive Oligomeric Proanthocyanidins

**DOI:** 10.3390/plants15030472

**Published:** 2026-02-03

**Authors:** Sarmite Janceva, Liga Petersone, Natalija Zaharova, Karina Schastnaja, Gints Rieksts, Anna Andersone

**Affiliations:** 1 Laboratory of Lignin Chemistry, Latvian State Institute of Wood Chemistry, Dzerbenes Street 27, LV-1006 Riga, Latvia; sarmite.janceva@kki.lv (S.J.); natalija.zaharova@gmail.com (N.Z.); karina.scastnaja@rpg.lv (K.S.); gints.rieksts@inbox.com (G.R.); 2 Department of Biochemistry, Riga Stradiņš University, Dzirciema Street 16, LV-1007 Riga, Latvia; liga.petersone@rsu.lv

**Keywords:** sea buckthorn, aronia, black currant, branches, extraction, oligomeric proanthocyanidins, mechanical processing, biomass pretreatment, biomass functionality composition, element analysis

## Abstract

This study aimed to evaluate the potential of branches of black chokeberry, sea buckthorn, and black currant as raw materials for the development of pharmacologically active compounds, primarily oligomeric proanthocyanidins (OPCs), as they exhibit a broad spectrum of biological activities, including antioxidant, antimicrobial, anti-inflammatory, anticancer, etc. Branch biomass collected in spring and autumn of 2023–2025 was analyzed for its functional group profile and used for the isolation of OPCs with ethanol, an ethanol–water mixture (1:1, *v*/*v*), and an ethanol–acetone–water mixture (4:1:5, *v*/*v*/*v*). The highest yield of OPCs (up to 14% of DB) was achieved using the ethanol–acetone–water solvent mixture. Using LC-MS/MS, the OPC composition was analyzed and found to consist of dimers (*m*/*z* 577), trimers (*m*/*z* 865), and tetramers (*m*/*z* 1153). The maximum OPC content was observed in autumn samples. Mechanical pretreatment enhanced OPC accessibility by disrupting cell walls and increasing particle surface, facilitating release from the matrix and yielding up to 1.2-fold more OPCs than from untreated biomass. Quantification of 22 elements in the biomass by ICP-MS revealed low levels of toxic metals along with the presence of nutritionally relevant elements. Therefore, from a chemical safety perspective, biomass can be considered suitable for use as a source of OPCs.

## 1. Introduction

Agro-industrial byproducts and residual biomass have attracted growing interest as sustainable sources of high-value-added bioactive compounds within the framework of circular economy strategies. Berry pomace, fruit peels, and seed cakes are rich in polyphenolic compounds exhibiting antioxidant, antimicrobial, and health-promoting properties, enabling their potential application in the food, nutraceutical, pharmaceutical, and cosmetic industries [1,2,3,4,5,6]. In contrast, pruning biomass generated from berry shrubs remains largely underexplored, despite its abundance and chemical richness. Therefore, the present study aims to evaluate the pruning biomass of sea buckthorn, black chokeberry, and black currant as a novel and sustainable source of oligomeric proanthocyanidins (OPCs), thereby expanding existing biomass valorization approaches toward higher added-value applications.

In recent years, growing attention has been directed toward the utilization of plant-derived byproducts and residual biomass as alternative sources of high-value-added bioactive compounds. Within agricultural and horticultural systems, woody residues, particularly the branches generated from berry shrub cultivation, represent a substantial yet largely underexploited biological resource. Looking at it from a bioeconomy perspective, the valorization of such biomass offers clear advantages, including improved resource efficiency, reduced waste streams, and a more sustainable use of renewable natural materials.

Proanthocyanidins (PACs), also known as condensed tannins, are ubiquitous polyphenolic secondary metabolites in plant tissues, well known for their pronounced biological activity [7]. PACs are formed through the condensation of flavan-3-ol monomers (have a typical C6–C3–C6 skeleton) [8,9] and represent the end products of the flavonoid biosynthesis pathway. PACs are also the second most abundant natural phenolic substances in nature, after lignin [10].

Depending on the degree of polymerization (DP), PACs are conventionally classified into oligomers and polymers. PACs with a DP of 2–4 are referred to as oligomeric PACs (OPCs), whereas those with a DP of 5 or higher are considered polymeric PACs (PPCs). The physicochemical and biological properties of PACs largely depend on their molecular structure, particularly on the degree of polymerization [11]. Dimeric and trimeric OPCs constitute the primary focus of PACs research, as they possess well-defined chemical structures and relatively high bioavailability. They are also more readily absorbed in the human gastrointestinal tract. In contrast, larger PPCs are not directly absorbed. Instead, some are excreted unchanged, while others undergo metabolism by the intestinal microbiota into smaller phenolic molecules, which may subsequently be absorbed and contribute to biological activity [11,12,13].

PACs exhibit a broad spectrum of biological effects, including antioxidant, anti-inflammatory, antimicrobial, and, potentially, anticancer properties [14]. Furthermore, many of them may have positive health effects on the central nervous system, thanks to their ability to cross the blood–brain barrier [15]. The beneficial impact of dietary PACs on the risk of cancer [16], cardiovascular diseases [17], and diabetes [18,19] is supported by many in vitro, animal, clinical, and epidemiological studies [20]. These multifunctional characteristics have driven sustained interest in PACs as ingredients in pharmaceutical formulations, functional foods, cosmetics, and emerging biomedical materials. Conventionally, PAC extraction has focused on fruits, seeds, or bark [21,22,23,24]. However, such raw materials are often seasonal, may compete directly with food production, and can present economic or logistical constraints.

Berry shrubs such as black chokeberry (*Aronia melanocarpa*, AR), black currant (*Ribes nigrum*, BC), and sea buckthorn (*Hippophae rhamnoides* L., SBT) are extensively cultivated across Northern Europe and the Baltic region on a significant commercial scale [25]. Routine pruning of these crops generates significant quantities of branch biomass each year, which is typically discarded or used only for low-value applications. Although woody plant tissues are known to contain substantial levels of polyphenols, including PACs, systematic data on the chemical composition and PAC recovery potential of berry shrub branch biomass remain scarce. As a result, berry shrubs are regarded primarily as sources of fruit, while the functional value of their vegetative parts remains largely unexplored.

According to the Central Statistical Bureau of Latvia [26], the combined cultivation area of SBT, BC, and AR increased from approximately 3057 ha in 2020 to about 4199 ha in 2024 (SBT = 1490 ha, BC = 2526 ha, and AR = 184 ha), highlighting the growing availability of pruning-derived biomass.

Our research on the vegetative parts of berry shrubs, initiated in 2022, has demonstrated that the branch extracts and their dominant polyphenols possess significant potential for application in cosmetics, pharmaceuticals, and related industries [27,28]. Long-term screening of berry shrub biomass with a focus on polyphenolic composition and its yearly changes is necessary for confirmation of their potential value as a raw material and contributes to the sustainable development of the horticultural sector, not only by increasing fruit yields, but also by enabling the production of high-value bioactive compounds such as PACs.

In this context, the aim of the present study was to evaluate the potential of AR, BC, and SBT branch biomass as an alternative source of OPCs based on the three-year studies. To achieve this, the extraction efficiency was investigated using different solvent systems and mechanical biomass pretreatment methods, followed by the quantitative determination of PAC content and characterization of PAC composition, functional group profile of the extracts, and elemental composition, which is important for valorizing branch biomass potential for the sustainable production of high-value-added bioactive PACs.

## 2. Results and Discussion

### 2.1. Qualitative and Quantitative Functional Analysis of Fruit Branches’ Biomass

The chemical composition of berry shrub branch biomass (AR, BC, SBT) was investigated using Fourier Transform Infrared Spectroscopy (FTIR) and functional quantitative wet chemistry analysis, revealing a range of functional group characteristics of lignocellulosic materials.

These included phenolic hydroxyl groups, carboxyl groups, acidic hydroxyl groups, aliphatic hydroxyl groups, and carbonyl groups. The quantitative distribution of these functional groups provides valuable insight into the chemical potential of the biomass for the recovery of bioactive compounds, including PACs and PAC-rich extracts, as well as for production of pharmaceutical excipients with antioxidative, antimicrobial, anticancer, and other biologically relevant properties.

**Figure 1:**
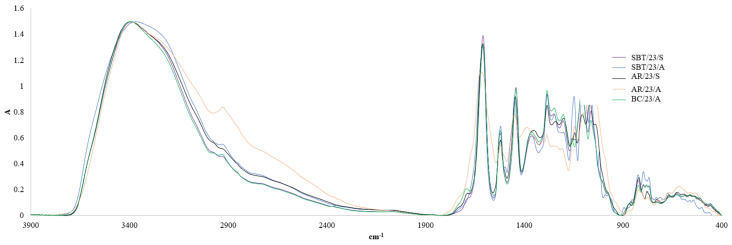
FTIR spectra of fruit shrub biomass collected in 2023.

**Figure 2:**
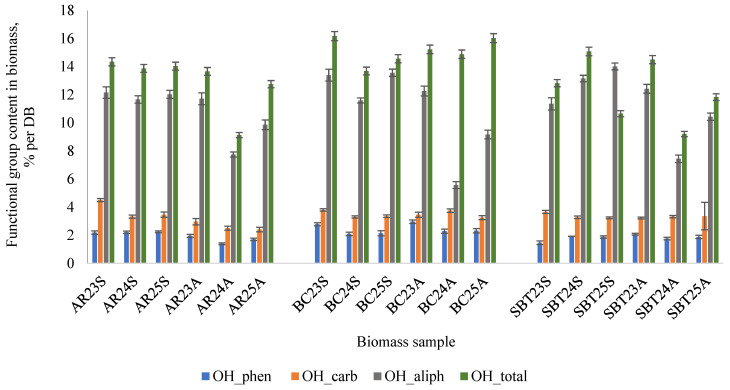
Functional group (OH_phen, OH_carb, OH_total, and OH_aliph) content in dry berry shrub branch biomass after crushing and homogenization: AR—aronia; BC—black currant; SBT—sea buckthorn; 23, 24, 25—biomass collection year, S—spring; A—autumn.

Phenolic hydroxyl groups (OH_phen), observed in the FTIR region of 3200–3550 cm^−1^ (Figure 1), are generally associated with polyphenolic constituents such as flavonoids, PACs, phenolic acids, and other phenolic compounds, and are directly linked to biological activity. The functional analysis revealed that the OH_phen content ranged from 1.5% to 2.9% per dry biomass (DB) (Figure 2).

Seasonal variations were noticeable in AR biomass, which exhibited slightly higher OH_phen values in spring compared to autumn. An elevated content of OH_phen groups has been reported in the literature [29,30] to be associated with increased antioxidant capacity, mainly due to their ability to donate hydrogen atoms and neutralize free radicals. Moreover, phenolic compounds rich in OH_phen groups have been linked to anti-inflammatory and antimicrobial activities in previous studies. Additionally, phenolic compounds (PACs) contribute to protein-binding properties, relevant for wound-healing formulations, gastroprotective agents, and natural preservatives [31].

Aliphatic hydroxyl groups (OH_aliph), which overlap with the broad OH absorption band around 3300–3500 cm^−1^, originate primarily from cellulose, hemicellulose, and other polysaccharide components. Their content ranged from 6 to 14% per DB (Figure 2), with higher values generally observed in spring-harvested biomass. The abundant OH_aliph groups in cellulose and hemicellulose are known to enhance hydrophilicity and contribute to the swelling capacity of polysaccharide-based materials, features that are particularly relevant for the formulation of pharmaceutical gels, films, and capsule materials. Furthermore, these OH_aliph groups provide reactive sites for chemical modification, such as esterification and etherification, which are widely used strategies to tailor biomass-derived materials for advanced biomaterial applications [32,33,34,35].

Carboxyl hydroxyl groups (OH_carb) were identified as a broadened shoulder in the 2700–3000 cm^−1^ region (Figure 1), characteristic of O-H stretching vibrations in carboxylic acids derived from organic acids or oxidized polysaccharides. Quantitative analysis showed OH_carb contents of 2–5% per DB, with the highest values observed in spring-harvested AR biomass (Figure 2). The presence of carboxyl groups increases material polarity and supports the formation of water-soluble fractions, making the biomass suitable for applications in pH-responsive polymers, gels, and stabilizers. In addition, carboxyl functionalities enable metal-ion complexation, enhancing sorption capacity and suggesting potential use in detoxification or heavy metal removal systems [36,37].

**Figure 3:**
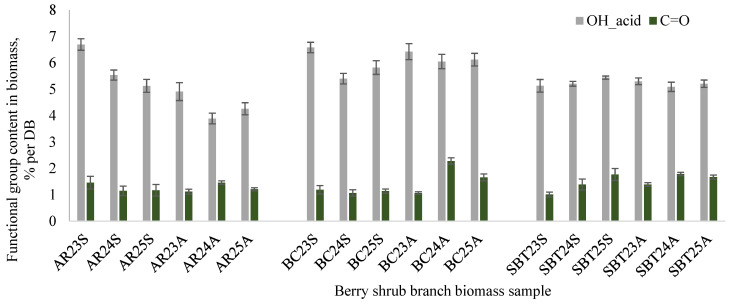
Functional group (OH_acid and C=O) content in dry berry shrub branch biomass after crushing and homogenization: AR—aronia; BC—black currant; SBT—sea buckthorn; 23, 24, 25—biomass collection year; S—spring; A—autumn.

Acidic hydroxyl groups (OH_acid), corresponding to the broad FTIR band between 2500 and 3000 cm^−1^ (Figure 1), were present at levels 4–7% per DB (Figure 3). According to literature data, OH_acid groups play a key role in determining the pH responsiveness of the biomass, its hydrogen-bonding capacity, and the stability of its extracts in polar solvents, which is crucial for pharmaceutical extraction and formulation. Furthermore, increased acidity may contribute to antimicrobial effects by creating conditions unfavorable for microbial growth and by influencing binding interactions when the biomass is used as an excipient or porous matrix [38,39,40,41].

The absorption band near 1700 cm^−1^ (Figure 1) corresponds to carbonyl group (C=O) stretching vibrations of C=O-containing structures, including esters, aldehydes, and carboxyl groups. In the analyzed samples, the carbonyl content was relatively low (1–2% per DB, Figure 3), indicating limited oxidation of polysaccharides. Such a low degree of oxidation is advantageous for maintaining chemical stability and preserving bioactive compounds during storage [42,43,44]. Nevertheless, C=O groups may also be associated with specific bioactive aldehydes.

Additional bands in the 1500–1600 cm^−1^ region (Figure 1) were assigned to aromatic C=C skeletal vibrations, confirming the presence of lignin and phenolic structures, including guaiacyl and syringyl units typical for hardwood lignin. The strong absorption region between 1000 and 1200 cm^−1^ reflects C-O and C-O-C stretching vibrations of polysaccharides, highlighting the dominance of cellulose and hemicellulose in the biomass matrix. The low fingerprint region (400–900 cm^−1^, Figure 1) further supports the presence of lignocellulosic components and phenolic derivatives through characteristic but less specific vibrational patterns.

Overall, the functional group composition and FTIR spectral profile indicate that berry shrub branch biomass represents a chemically diverse and promising raw material for pharmaceutical and biotechnological applications. The combination of phenolic hydroxyl groups, aliphatic alcohols, carboxyl functionalities, aromatic lignin structures, and abundant polysaccharides supports its potential use in the production of natural antioxidants containing PACs, the formulation of bioactive extracts, sorbents, excipients, and the development of biomaterials. However, further detailed compositional analysis and comprehensive bioactivity testing are required to fully assess its industrial applicability.

Based on the quantitative functional analysis data (Figure 2) on the content of phenolic and aliphatic hydroxyl groups, all analyzed biomass samples are suitable for the extraction of oligomeric polyphenols–PACs. Nevertheless, based solely on the functional group data, it is not possible to determine which season or biomass variety is most suitable for the extraction of the target compounds. Therefore, the biomass was extracted using different solvent systems to enable a quantitative analysis of PACs in the obtained extracts.

### 2.2. Assessment of Fruit Branch Biomass as a Source of PACs

**Figure 4:**
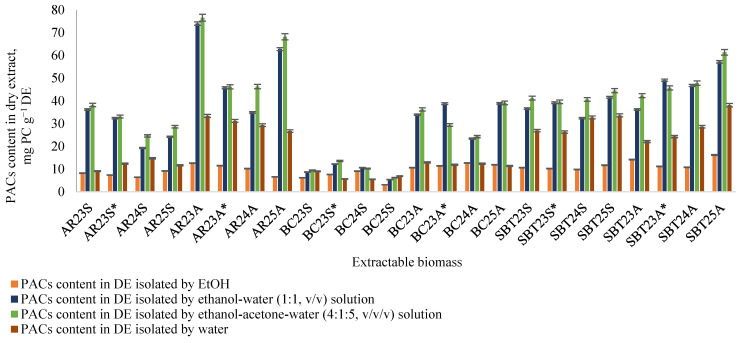
PAC content in extracts isolated from fruit branch biomass: AR—aronia; BC—black currant; SBT—sea buckthorn; 23, 24, 25—biomass collection year; S—spring; A—autumn; * biomass collected in Aizkraukle.

During the extraction of fruit branch biomass using varying numbers of consecutive 30 min extraction cycles, a series of extracts was obtained and analyzed for PAC content using the butanol–HCl method (Section 3.6). The inclusion of 10% acetone in the ethanol–water extraction solvent (ethanol–acetone–water, 4:1:5, *v*/*v*/*v*) improved PAC yield and quantification accuracy. The relative content of soluble PACs varied considerably among the investigated biomass types. Analysis of the dry extracts (DEs) revealed that the highest PAC concentrations were detected in AR and SBT branch samples extracted with the ethanol–acetone–water mixture (4:1:5, *v*/*v*/*v*). Specifically, AR samples (AR23A, AR24R, AR25R) contained 46.3–76.7 mg PC∙g^−1^ DE), while SBT samples (SBT23A, SBT24A, SBT25A) ranged from 42 to 61 mg PC∙g^−1^ DE) (Figure 4). The ethanol–water solvent system (1:1, *v*/*v*) represented the second most efficient extraction medium, yielding comparable but slightly lower concentrations, as exemplified by AR23A (74.0 mg PC∙g^−1^ DE) and SBT25A (57.2 mg PC∙g^−1^ DE) (Figure 4).

**Figure 5:**
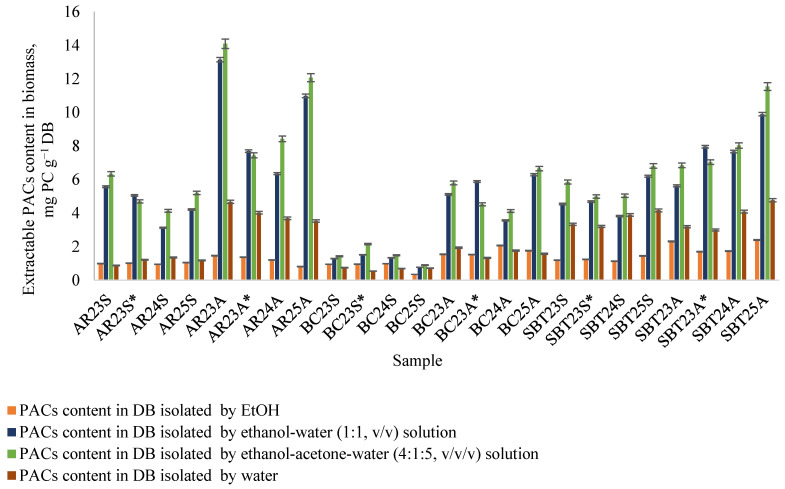
Isolated PAC yield from fruit branch biomass. AR—aronia; BC—black currant; SBT—sea buckthorn; 23, 24, 25—biomass collection year; S—spring; A—autumn; * biomass collected in Aizkraukle.

In 2023, biomass was collected from two different plantations (see Section 3.1). The extraction yields, PAC contents in the extracts obtained using three different solvent systems, and PAC yields from the biomass are shown in Figure 5, confirming that the parameters depend on the place of growth.

When the ethanol–acetone–water mixture (4:1:5, *v*/*v*/*v*) was applied, the overall PACs yield from branch biomass ranged from 1 to 14% per DB (Figure 5). A pronounced seasonal effect was observed: biomass collected in autumn consistently exhibited significantly higher PAC levels than spring-collected samples. This observation aligns well with the existing literature describing seasonal accumulation of polyphenols in woody tissues during the later stages of vegetative development [45]. Collectively, these findings demonstrate that both solvent composition and the applied analytical protocol exert a decisive influence on the assessment of PAC content. They further emphasize the necessity of methodological standardization when comparing PAC yields across different biomass types, harvest seasons, and extraction strategies.

### 2.3. Isolated PAC Mixture Characterization

In recent years, LC-DAD-ESI-MS/MS has emerged as a cornerstone technique for the detailed characterization of PACs. Its principal advantage lies in the rapid and highly accurate determination of molecular weights, coupled with the generation of relatively few fragment ions during the analysis of polymeric structures. This feature substantially diminishes the reliance on reference standards, facilitating more streamlined investigations.

Nevertheless, a persistent limitation of ESI-MS is the gradual decrease in signal intensity and detection sensitivity as the degree of polymerization (DP) increases. This phenomenon poses a significant challenge, particularly when attempting to identify PACs present at low concentrations within complex mixtures [46].

**Figure 6:**
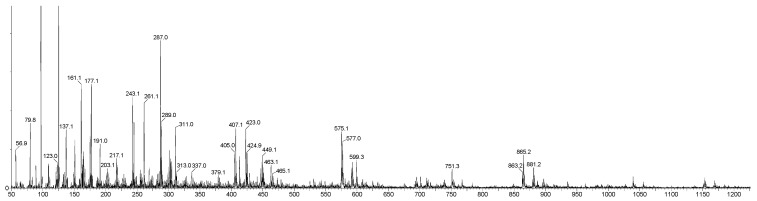
MS spectra of purified PAC samples from extract isolated from BC24A by ethanol–acetone–water solution (4:1:5, *v*/*v*/*v*).

**Figure 7:**
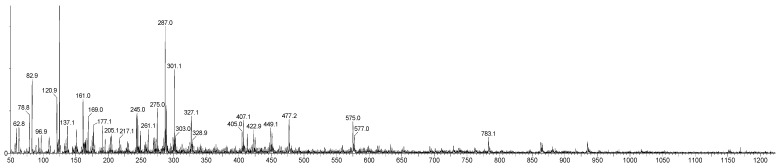
MS spectra of purified PAC samples from extract isolated from SBT24A by ethanol–acetone–water solution (4:1:5, *v*/*v*/*v*).

**Figure 8:**
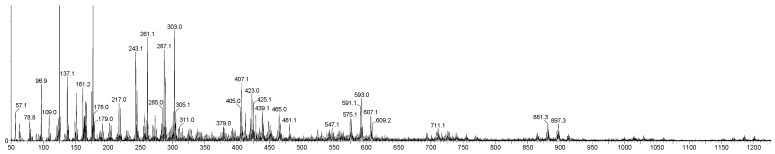
MS spectra of purified PAC samples from extract isolated from AR24A by ethanol–acetone–water solution (4:1:5, *v*/*v*/*v*).

Consequently, the present study concentrated on the detection and structural elucidation of OPCs with DP values up to 4. Analyses conducted in negative ion mode revealed pronounced [M-H]^−^ ion peaks, corresponding to B-type procyanidin dimer (DP2, *m*/*z* 577), trimer (DP3, *m*/*z* 865), and tetramer (DP4, *m*/*z* 1153). Literature reports indicate that the monomeric units, catechins (C) and epicatechin (EC), yield characteristic fragment ions at *m*/*z* 245, 205, 179, and 125, respectively [24]. Moreover, fragmentation of the procyanidin trimer (*m*/*z* 577) generates diagnostic ions at *m*/*z* 451, 425, 407, and 289, providing essential fingerprints for structural identifications [47]. The LC-DAD-ESI-MS/MS spectra of purified PACs samples from AR24A, SBT24A, and BC24A are shown in Figure 6, Figure 7 and Figure 8.

The composition of PACs isolated from AR, SBT, and BC biomass was investigated for a second time, but for the 2024 cultivation year, and for the first time for BC. The PACs isolated from AR24A, SBT24A, and BC24A extracts consisted of a complex mixture of B-type procyanidin dimers (*m*/*z* 577), creating fragment ions at *m*/*z* 407.07; 289.07; 245.08; 202.08; 161.02; 125.02, trimers (*m*/*z* 865), creating fragment ions at *m*/*z* 695.15; 577.14; 451.11; 407.08; 289.07, and tetramers (*m*/*z* 1153), creating fragment ions at *m*/*z* 577.13; 525.08; 449.09; 407.08; 287.06; 243.03; 161.02. These results (Figure 6, Figure 7 and Figure 8) confirmed previously investigated for the year 2021 [28] and confirmed that dimers, trimers, and tetramers have higher relative abundance in the purified PAC fractions.

### 2.4. Assessment of Insoluble PACs in Biomass Composition

Insoluble PACs in the biomass were quantified following the extraction process, revealing contents in the residual material ranging from 0.4 to 1.3% per DB. Notably, the lowest residual PACs content (0.4% per DB) was detected after extraction using an ethanol–acetone–water mixture, underscoring the remarkable efficiency of this solvent system in recovering both monomeric and oligomeric PACs forms. Nevertheless, these findings also highlight that a considerable portion of PACs remains tightly bound within the plant matrix, suggesting that mechanical processing is essential to improve their accessibility and facilitate extraction [48]. Techniques such as ball milling or mortar grinding have gained attention as promising, solvent-free strategies that act directly on the biomass [49]. By disrupting cell wall structures and significantly increasing surface area, these mechanical treatments promote the release of matrix-bound PACs, enabling their interaction with digestive fluids without relying on chemical solvents.

From a nutritional standpoint, retaining PACs within the intact biomass may offer distinct advantages. Polymeric PACs predominantly exert their biological activity in the colon, where they interact with the gut microbiota, rather than being absorbed systemically. Consequently, preserving the polymeric forms within the whole plant material may enhance their health-promoting effects in situ, providing a functional benefit beyond mere extraction efficiency.

### 2.5. Influence of Mechanical Processing on PAC Isolation from Biomass

**Figure 9:**
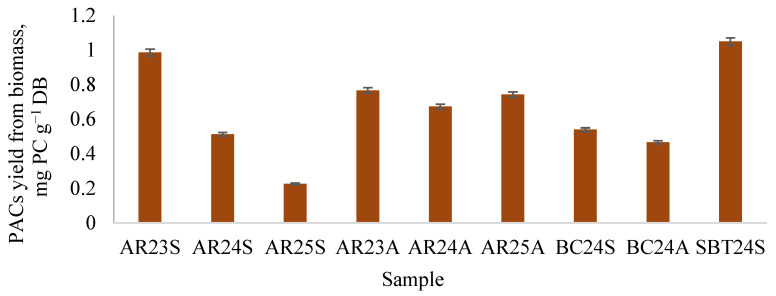
Difference in PAC yield as a result of Retch mechanical mortar processing prior to extraction with an ethanol/acetone/water mixture (4:1:5, *v*/*v*/*v*).

Mechanical processing of biomass (AR, BC, SBT) was performed using a Retch mechanical mortar, which ground the samples through continuous rubbing for 20 min at a rotational speed of 100 rpm. This treatment effectively disrupted structural and intermolecular bonds, significantly enhancing the accessibility of PACs. The effect of this mechanical intervention was confirmed experimentally: PACs extracted from the processed biomass exhibited up to a 1.2-fold increase in yield compared to untreated samples (Figure 9).

These findings suggest that biomass with particle sizes up to 0.5 mm can be employed directly as pharmacological or nutraceutical material, eliminating the need for additional extraction steps. The Retch mortar demonstrated high efficiency not only in reducing particle size but also in breaking down cellular structures, thereby increasing surface area and porosity [49,50]. Nitrogen adsorption–desorption analysis of the mechanically treated dominant sample (AR24A) revealed a specific surface area of 0.664 m^2^ g^−1^ and a total pore volume of 1.66 mm^3^ g^−1^, markedly higher than the initial biomass (0.51 m^2^ g^−1^ and 0.72 mm^3^ g^−1^, respectively). This enhanced microstructure facilitates direct accessibility of bioactive compounds, supporting either improved extractability or potential biological activity of PACs without further chemical extraction.

### 2.6. Element Analysis of Fruit Branches’ Biomass

Elemental profiling of biomass samples (AR24S, AR24A, BC24S, BC24A, SBT24S) after mechanical pretreatment (MP) revealed substantial amounts of essential macro- and microelements, including calcium (Ca), magnesium (Mg), iron (Fe), copper (Cu), zinc (Zn), and manganese (Mn), which contribute to dietary mineral intake. Potentially toxic elements such as mercury (Hg), cadmium (Cd), thallium (Tl), and antimony (Sb) were below detection limits (<0.002 mg∙kg^−1^ for Hg, <0.0004 mg∙kg^−1^ for Cd, <0.01 mg∙kg^−1^ for Tl and Sb), indicating minimal risk. Lead (Pb) concentration ranged from 0.30 to 0.59 mg∙kg^−1^, necessitating comparison with regulatory thresholds (for instance, EU limits for fruits at 0.1–0.3 mg∙kg^−1^ [51]). Arsenic (As) ranged from 0.02 to 0.06 mg∙kg^−1^, remaining well within acceptable limits. Chromium (Cr) and nickel (Ni) levels were highest in BC biomass (0.82 mg∙kg^−1^ for Cd, 0.17–0.14 mg∙kg^−1^ for Ni) but still below toxic thresholds for human consumption. Overall, the biomass exhibits low levels of toxic metals while providing nutritionally relevant elements, supporting its suitability for dietary applications and as a reliable source of PACs (Table 1).

Based on Commission regulation (EU) 2023/915, the content of heavy metals does not exceed the permissible limits [51], indicating that the AR, BC and SBT biomass, even without extraction, can be used as a source of PACs.

## 3. Materials and Methods

### 3.1. Material Collection and Preparation

The branches of SBT, cultivar ‘Maria Bruvele’, were collected from the SBT plantation in Seme parish, Tukums county of Latvia (DD: 57.1444093, 23.108156). BC, cultivar ‘Selechenskaja’, and AR, cultivar ‘Mulatka’, were collected from the fruit shrub plantation in Baldone parish, Kekava county of Latvia (Decimal degrees (DD): 56.82065, 24.27653). For comparison, the branches of SBT, AR and BC were also collected from the fruit shrub garden in Aizkraukle, Latvia (DD: 56.595200, 25.243633) in 2023.

**Figure 10:**
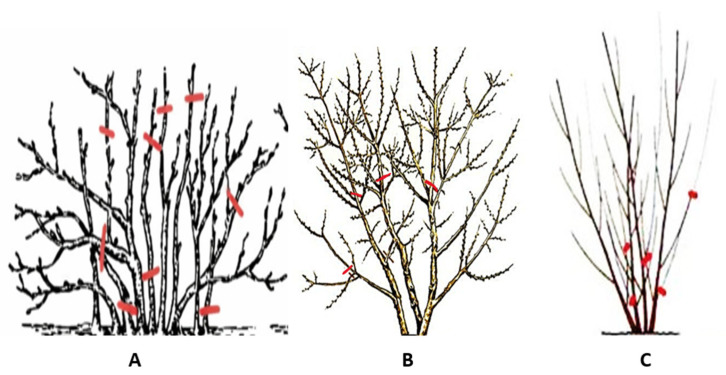
Scheme of pruning bushes for biomass collection: (**A**) BC; (**B**) SBT; (**C**) AR. The red line indicates the place of cutting.

The collected branches with different branch diameters (1–8 cm), following the pruning scheme (Figure 10), were chipped directly at the sampling site to produce chips not exceeding 5 cm in size. For further analysis, the chips were dried at 20–25 °C and ground using a rotary mill (Cutting Mill SM100, Retsch, Haan, Germany). The resulting ground material (hereinafter referred to as biomass) was stored at −8 °C in airtight containers until analysis. The biomass sample abbreviations are provided in Table 2.

### 3.2. Chemicals

Procyanidin B2, Sephadex LH-20, butanol, formic acid, potasium bromide (KBr), nitric acid, acetonitrile, acetone, ethanol, ferric ammonium sulfate dodecahydrate (FeNH_4_(SO_4_)_2_∙12H_2_O), acetic ahydride (Ac_2_O), lithium hydroxide (LiOH), sodium hydroxide (NaOH), pyridine, hydrochloric acid (HCl), ICP multi-element standard solution XVI (21 elements: Sb; As; Be; Cd; Ca; Cr; Co; Cu; Fe; Pb; Li; Mg; Mn; Mo; Ni; Se; Sr; Tl; Ti; V; Zn and ICP multi-element standard solution IX (9 elements: As; Be; Cd; Cr(VI); Hg; Ni; Pb; Se; Tl were obtained from Merck (Darmstadt, Germany).

### 3.3. FTIR Analysis

FTIR spectra of the biomass samples were recorded in KBr pellets (1.6 mg of the sample in 200 mg of KBr (IR grade, Sigma-Aldrich, Inc., St. Louis, MO, USA), in the range of 4400–400 cm^−1^ (resolution of 4 cm^−1^, 32 scans), using a Nicolet iS50 FT-IR spectrometer (Thermo Scientific, Waltham, MA, USA) [52]. The Spectrum v5.0.1 program was used for processing the spectrum.

### 3.4. Determination of the Number of Functional Groups in the Extract

The functional groups (carboxyl –OH_carb and phenolic –OH_phen) in the extracts were determined by acid-base conductometric titration (InoLab Level 3, WTW GmbH & Co. KG, Weilheim, Germany), according to the method described by Zakis [53]. When an alkali is introduced into the titration medium (direct titration), the degree of ionization of acidic groups increases, resulting in a rise in the overall electrical conductivity of the system. Conversely, when the same system is titrated with an acid (back titration), the conductivity decreases. Depending on the acidity strength, the less acidic phenolic –OH_acid_ groups are neutralized first, followed by the more strongly acidic carboxyl –OH_carb_ groups. Approximately 50 mg of the extract was weighed into a 10 mL nitrogen-flushed vial, and 5 mL of 0.1 M NaOH solution was added. After 24 h, the mixture was quantitatively transferred with distilled water into a titration vessel. A conductometric cell (electrodes) was placed into the solution, and the titration was carried out using 0.1 M HCl until a total volume of approximately 6 mL was reached. The titration process was controlled and recorded in a computerized mode.

The content of carboxyl and phenolic hydroxyl groups was calculated using Formulas (1) and (2):

(1) $$OH\_carb=\frac{a\cdot 1.7}{A}\cdot 100\%$$

(2) $$OH\_phen=\frac{b\cdot 1.7}{A}\cdot 100\%$$

where

OH_carb—the content of carboxyl groups in the sample, % per DB;
OH_phen—the content of phenolic hydroxyl groups in the sample, % per DB;
a,b—consumed titrant volume, mL;
A—dry weight mass of extract (moisture < 1%), mg;
1.7—a mass of hydroxyl groups corresponding to 1 mL of 0.1 M HCl solution, mg.

Total OH groups content in biomass (OH_total = OH_phen + OH_aliph) was determined by the method of acetylation [53]. The test tube was weighed, the biomass sample in the amount of 20 mg was placed into a test tube with a stopper, and the test tube with biomass was weighed once again to determine the precise biomass sample weight. The acetylation reagent in the amount of 0.1 mL (a mix of acetic anhydride with pyridine, prepared exactly before the analysis, with 4.7 mL of Ac_2_O and 4 mL of pyridine) was added to the test tube, and the test tube was weighed again to determine the precise mass of acetylation reagent. The test tube was placed on the tripod under pressure and kept at 50 °C for 24 h; after that, with addition of 10 mL of acetone was transferred to the titration vessel, and a necessary amount of destilled water was added, and then titrated with 0.1 N LiOH for potentiometric titration. The amount of the OH_total is determined by Formula (3):

(3) $$OH\_total=\frac{(a\frac{b_{0}}{a_{0}}-b)\cdot f\cdot 1.7}{G}\cdot 100,\;\%$$

where

OH_total—the content of total hydroxyl groups in the sample, % per DB;
a_0_—acetylation mix mass in control experiment, mg (average between 3 repetitions);
a—acetylation reagent mass, mg;
b—0.1 N LiOH volume used in titration of the reaction mixture after acetylation, mL;
b_0_—0.1 N LiOH volume used in titration of the control sample, mL;
f—0.1 N LiOH factor;
G—a mass of the analyzed sample, mg;
1.7—a mass of hydroxyl groups corresponding to 1 mL of 0.1 M HCl solution, mg.

### 3.5. Biomass Extraction

Milled biomass samples (n = 24) with a particle size of 1–2 mm were used for extraction. Biomass extraction was performed using different solvents: ethanol, distilled water, aqueous ethanol solution (1:1, *v*/*v*), and an ethanol/acetone/water mixture (4:1:5, *v*/*v*/*v*). The ratio of dry biomass to solvent was 1:8 (*w*/*v*). Extraction was carried out in a flask for 30 min per cycle, using two consecutive cycles, under heating and continuous stirring with a magnetic stirrer while maintaining the temperature at 60 ± 5 °C. After extraction, ethanol and acetone were removed by vacuum distillation at 60 ± 2 °C using a Heidolph Hei-VAP G3 All-round Chill Package (Heidolph Instruments GmbH&Co. KG, Germany), and the resulting aqueous phase was cooled to 25 ± 2 °C. Following cooling, the aqueous extracts were deep-frozen and subsequently placed in a lyophilization chamber Heto PowerDry PL 300 (Thermo Scientific, Waltham, MA, USA), where, under vacuum conditions (1.2–1.5 hPa) and low temperature (−50 °C), the frozen solvent was removed by sublimation. As a result, dry homogeneous, powdery extracts were obtained for further analysis.

### 3.6. Determination of the PACs in the Extracts and Biomass

The method for quantification of PACs by butanol-HCl assay was performed according to Porter et al. [54], with procyanidin dimer B2 used as a reference compound. In a heated alcoholic medium (butanol) with the presence of a strong acid (HCl), the interflavan bonds of PACs are disrupted, forming flavanyl carbocations which rapidly evolve to anthocyanidins, leading to a pink/red color measurable at 550 nm according to a standard curve. In 10 mL glass tubes, 6 mL of butanol–HCl (95:5, *v*/*v*) and 0.2 mL of 2% FeNH_4_(SO_4_)_2_∙12 H_2_O (*w*/*v*) in 2 N HCl were added to 1 mL of the extract aliquots. The tubes were closed hermetically with caps equipped with a Teflon seal and heated at 80 °C for 50 min in a dry bath. After cooling, samples were filtered through a Teflon membrane (0.45 µm, Interchim, Montluçon, France) before analysis. Absorbance at 550 nm was measured using a UV/VIS spectrometer Lambda 650 (Perkin Elmer, Shelton, CT, USA). The content of PACs was expressed in grams of PC equivalent per gram of DE (mg PC g^−1^ DE). All experiments were performed in triplicate.

The content of PACs soluble in ethanol/ethanol–acetone–water/water in the biomass samples was calculated based on the extraction yield and PACs concentration determined in the corresponding extract. The total PAC content of the biomass sample was determined directly in the biomass itself rather than in an extract, using biomass with a particle size ≤ 2 mm. All experiments were performed in triplicate.

### 3.7. PAC Isolation for Characterization

PACs were isolated from the extracts by column chromatography. The obtained extracts by ethanol/acetone/water mixture were applied to a Sephadex LH-20 column (2.5 cm × 120 cm) and successively eluted with ethanol followed by aqueous acetone (70%, *v*/*v*) at room temperature. The elution scheme was selected based on a previous study, with minor modifications. Each extract yielded two fractions, which were lyophilized by Heto PowerDry PL 300 (Thermo Scientific, Waltham, MA, USA) after removal of the organic solvents.

### 3.8. PAC Composition Determination

Isolated PACs were dissolved in aqueous methanol (*v*/*v* 20:80) to an approximate concentration of 0.1 mg∙mL^−1^, filtered, and subsequently analyzed by MS/MS chromatography. The MS spectra were recorded using a Waters Acquity UPLC H-Class system equipped with a PDA detector and coupled to a Micromass Quattro Micro mass spectrometer (Waters Corp., Milford, MA, USA). Chromatographic separation was performed in an Acquity UPLC BEH Amide column (1.7 μm, 3.0 × 100 mm). The mass spectrometer was operated in negative electrospray ionization (ESI^−^) mode with a cone voltage of −40 V, using direct infusion. The source and desolvation temperatures were maintained at 130 and 300 °C, respectively, while the cone and desolvation nitrogen gas flow rates were set as 96 and 395 L∙h^−1^.

The mobile phase consisted of 0.1% formic acid, water (A), and acetonitrile (B), with a flow rate of 0.35 mL∙min^−1^ under the gradient program of 5–20% (B) for an initial 1 min, 20–25% (B); 5–6 min, 25–75% (B), 6–7 min, 75–80% (B), 7–8 min, 80–5% (B), 8–10 min, 5% (B); the injection volume was 2.0 μL.

### 3.9. Mechanical Processing of Biomass

**Figure 11:**
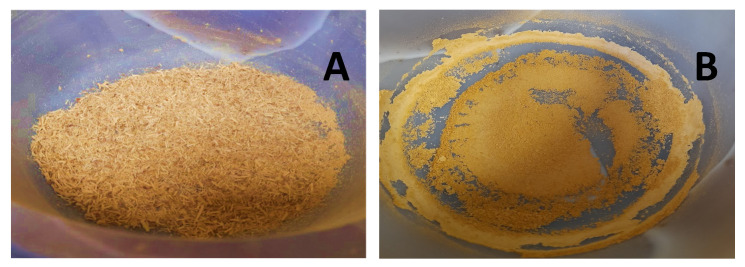
SBT biomass before (**A**) and after mechanical treatment (**B**).

Mechanical treatment (MT) of biomass was carried out separately for each sample in an original trituration-typemill (RM 200, Retsch, Germany). The trituration of biomass was carried out for 20 min at 100 rpm (Figure 11).

### 3.10. Extraction of Mechanically Treated Biomass

Mechanically treated biomass (MTB) samples (n = 5, 3 repetitions) with a particle size up to 0.5 mm were used for extraction. MTB extraction was performed using an ethanol/acetone/water mixture (4:1:5, *v*/*v*/*v*). The ratio of dry biomass to solvent was 1:8 (*w*/*v*). Extraction was carried out in a flask for 30 min per cycle, using two consecutive cycles, under heating and continuous stirring with a magnetic stirrer while maintaining the temperature at 60 ± 5 °C. Extraction of the extracts in powder form, including solvent evaporation and lyophilization, is described in Section 3.5.

### 3.11. Inorganic Element Quantification by ICP-MS

#### 3.11.1. Sample Preparation

A 0.50 g portion of biomass was placed into Teflon digestion vessels. For inorganic elemental analysis, the samples were digested using microwaves within a closed system (CEM MARS 6, Mathews, NC, USA) at 200 °C using 6 mL of HNO_3_ and 2 mL of H_2_O_2_. After cooling, the digests were transferred to volumetric flasks and diluted to a final volume of 50 mL with deionized water, followed by filtration through a 0.2 μm PTFE glass fiber filter (Agilent Technologies, Santa Clara, CA, USA). All samples were prepared and analyzed in triplicate.

#### 3.11.2. Inorganic Element Quantification

An ICP-MS system (iCAP TQe, Thermo Fisher Scientific GmbH, Bremen, Germany) equipped with a PFA nebulizer and coupled to an ASX-560 autosampler (Thermo Fisher Scientific GmbH, Bremen, Germany) was used to determine the concentrations of 30 elements in the samples. The plasma power was set to 1549 w, with an argon plasma gas flow rate of 14 mL/min, and an auxiliary gas flow of 0.8 mL/min. The peristaltic pump speed of the ICP-MS was set to 70 rpm. The instrument control acquisition was performed using Qtegra™ software version 2.11.

The tuning procedure was performed daily using a multielement solution containing Ba, Bi, Ce, Co, Ho, In, Li, Mg, Ti, U, and Y (1.0 µL/L each). External calibration was performed using multielement standard solutions: ICP multi-element standard solution XVI (21 elements: Sb; As; Be; Cd; Ca; Cr; Co; Cu; Fe; Pb; Li; Mg; Mn; Mo; Ni; Se; Sr; Tl; Ti; V; Zn). Calibration standards were prepared in 2% nitric acid (*v*/*v*) at the following concentration levels: 0, 0.4, 1.3, 3.8, 11.4, 34.3, 102.9, 308.6, 925.9, 25,000 µg∙L^−1^.

### 3.12. Statistical Analysis

All experiments had 3 repetitions. The results were expressed as means. Statistical analyses were performed using Microsoft Excel 2016. Confidence intervals (CI) for a mean using Student’s T distribution were calculated at a significance level of 5% (α = 0.05).

## 4. Conclusions

The combined FTIR and quantitative functional group analysis demonstrates that berry shrub branch biomass possesses a chemically diverse and functionally rich composition. The presence of substantial amounts of phenolic hydroxyl, aliphatic hydroxyl, carboxyl, acidic, and carbonyl groups reflects a balanced lignocellulosic-polyphenolic matrix with pronounced biological and technological potential. Elevated levels of phenolic and carboxyl hydroxyl groups, particularly in spring-harvested biomass, indicate strong antioxidant, antimicrobial, and protein-interacting capabilities, while the abundance of aliphatic hydroxyl groups highlights favorable hydrophilicity. Together, these features support the use of berry shrub branch biomass as a promising raw material for the development of bioactive extracts, pharmaceutical excipients, sorbents, and functional biomaterials. Nevertheless, further targeted compositional characterization and comprehensive bioactivity studies are required to fully translate this potential into industrial and biomedical applications.

Berry shrub (AR, SBT, BC) branch biomass represents a promising source of PACs. The highest PAC yield was obtained using an ethanol–acetone–water (4:1:5, *v*/*v*/*v*) extraction, with autumn-collected biomass containing higher levels than spring-collected samples. LC-DAD-ESI-MS/MS analysis characterized B-type dimers, trimers, and tetramers, while mechanical processing enhanced the accessibility of both soluble and insoluble PACs. Retaining polymeric PACs within the intact biomass may enhance their biological activity in the colon. Mechanical processing of biomass using Retch mortar effectively disrupts cellular structures and intermolecular bonds, enhancing PACs accessibility and increasing their yield up to 1.2 times compared to untreated biomass. Biomass with particle size up to 0.5 mm can be used directly for pharmacological or nutraceutical purposes without additional extraction. Elemental analysis confirmed high levels of essential nutrients and low levels of toxic metals, supporting the safety and nutritional value of the biomass as a reliable source of PACs.

## Figures and Tables

**Table 1:** Elemental analysis of mechanically treated biomass collected in spring and autumn 2024.

Element	AR24S/MP	AR24A/MP	BC24S/MP	BC24A/MP	SBT24S/MP
	Element Concentration, mg∙kg^−1^				
Be	0.08 ± 0.03	0.09 ± 0.03	0.09 ± 0.03	0.10 ± 0.02	0.09 ± 0.03
Hg *	˂0.002	˂0.002	˂0.002	˂0.002	˂0.002
Cr	0.05 ± 0.01	0.24 ± 0.03	0.82 ± 0.05	0.20 ± 0.02	0.07 ± 0.03
Ni	0.14 ± 0.03	0.12 ± 0.03	0.14 ± 0.03	0.17 ± 0.03	0.10 ± 0.03
As	0.04 ± 0.02	0.03 ± 0.02	0.04 ± 0.02	0.06 ± 0.03	0.02 ± 0.01
Se	0.02 ± 0.01	0.02 ± 0.01	0.03± 0.01	0.02 ± 0.01	0.03 ± 0.01
Cd *	˂0.0004	˂0.0004	˂0.0004	˂0.0004	˂0.0004
Pb	0.47 ± 0.05	0.59 ± 0.03	0.55 ± 0.03	0.32 ± 0.05	0.30 ± 0.05
Tl *	˂0.01	˂0.01	˂0.01	˂0.01	˂0.01
Sb *	˂0.01	˂0.01	˂0.01	˂0.01	˂0.01
Ca	453.9 ± 0.2	679 ± 12	811 ± 7	751 ± 9	359 ± 10
Cu	2.1 ± 0.1	4.2 ± 0.1	5.1 ± 0.1	3.4 ± 0.3	3.0 ±0.4
Fe	30 ± 3	33 ± 1	35± 2	47 ± 6	14 ± 4
Li	0.11 ± 0.04	0.09 ± 0.05	0.08 ± 0.05	0.10 ± 0.02	0.04 ± 0.01
Mg	458 ± 11	604 ± 46	1104 ± 41	1028 ± 49	188 ± 7
Mn	4.6 ± 0.4	5.3 ± 0.3	19.5 ± 0.5	15.1 ± 0.2	6.5 ± 0.5
Mo	0.08 ± 0.01	0.06 ± 0.02	0.38 ± 0.03	0.34 ± 0.05	0.80 ± 0.04
Zn	8.1 ± 0.5	13.0 ± 0.1	13.4 ± 0.1	15.2 ±0.2	4.9 ± 0.5
Sr	8 ± 2	10 ± 2	15 ± 1	14 ± 1	10 ± 2
Ti	2.4 ± 0.5	2.3 ± 0.2	2.4 ± 0.2	2.7 ± 0.3	0.5 ± 0.1
V	0.08 ± 0.02	0.06 ± 0.02	0.07 ± 0.03	0.13 ±0.04	≤ 0.01
Co	0.02 ± 0.01	0.02 ± 0.01	0.02 ± 0.01	0.02 ± 0.01	˂0.01

* Below the quantification limit.

**Table 2:** Abbreviation of biomass samples.

Sample Abbreviation	Collection Year, Month Period	Sample Abbreviation	Collection Year, Month Period	Sample Abbreviation	Collection Year, Month Period
Black chokeberry (*Aronia melanocarpa*)		Blackcurrant (*Ribes nigrum*)		Sea buckthorn (*Hippophae rhamnoides* L.)	
AR23S	2023, March	BC23S	2023, March	SBT23S	2023, March
AR24S	2024, March	BC24S	2024, March	SBT24S	2024, March
AR25S	2025, March	BC25S	2025, March	SBT25S	2025, March
AR23A	2023, September	BC23A	2023, September	SBT23A	2023, September
AR24A	2024, September	BC24A	2024, September	SBT24A	2024, September
AR25A	2025, September	BC25A	2025, September	SBT25A	2025, September
AR23S *	2023, March	BC23S *	2023, March	SBT23S *	2023, March
AR23A *	2023, September	BC23A *	2023, September	SBT23A *	2023, September

* from Aizkraukle.

## Data Availability

Data are contained within the article.

## References

[1] Karastergiou A., Gancel A.-L., Jourdes M., Teissedre P.-L. (2024). Valorization of Grape Pomace: A Review of Phenolic Composition, Bioactivity, and Therapeutic Potential. Antioxidants.

[2] Mirabella N., Castellani V., Sala S. (2014). Current Options for the Valorization of Food Manufacturing Waste: A Review. J. Clean. Prod..

[3] Sagar N.A., Pareek S., Sharma S., Yahia E.M., Lobo M.G. (2018). Fruit and Vegetable Waste: Bioactive Compounds, Their Extraction, and Possible Utilization. Comp. Rev. Food Sci. Food Safe.

[4] Meremäe K., Raudsepp P., Rusalepp L., Anton D., Bleive U., Roasto M. (2024). In Vitro Antibacterial and Antioxidative Activity and Polyphenolic Profile of the Extracts of Chokeberry, Blackcurrant, and Rowan Berries and Their Pomaces. Foods.

[5] Pedisić S., Zorić Z., Repajić M., Levaj B., Dobrinčić A., Balbino S., Čošić Z., Dragović-Uzelac V., Elez Garofulić I. (2025). Valorization of Berry Fruit By-Products: Bioactive Compounds, Extraction, Health Benefits, Encapsulation and Food Applications. Foods.

[6] Dienaitė L., Pukalskas A., Pukalskienė M., Pereira C.V., Matias A.A., Venskutonis P.R. (2020). Phytochemical Composition, Antioxidant and Antiproliferative Activities of Defatted Sea Buckthorn (*Hippophaë rhamnoides* L.) Berry Pomace Fractions Consecutively Recovered by Pressurized Ethanol and Water. Antioxidants.

[7] Prior R.L., Lazarus S.A., Cao G., Muccitelli H., Hammerstone J.F. (2001). Identification of Procyanidins and Anthocyanins in Blueberries and Cranberries (*Vaccinium* Spp.) Using High-Performance Liquid Chromatography/Mass Spectrometry. J. Agric. Food Chem..

[8] Nijveldt R.J., Van Nood E., Van Hoorn D.E., Boelens P.G., Van Norren K., Van Leeuwen P.A. (2001). Flavonoids: A Review of Probable Mechanisms of Action and Potential Applications. Am. J. Clin. Nutr..

[9] Calderaro A., Patanè G.T., Tellone E., Barreca D., Ficarra S., Misiti F., Laganà G. (2022). The Neuroprotective Potentiality of Flavonoids on Alzheimer’s Disease. Int. J. Mol. Sci..

[10] Yu K., Song Y., Lin J., Dixon R.A. (2023). The Complexities of Proanthocyanidin Biosynthesis and Its Regulation in Plants. Plant Commun..

[11] Nie F., Liu L., Cui J., Zhao Y., Zhang D., Zhou D., Wu J., Li B., Wang T., Li M. (2023). Oligomeric Proanthocyanidins: An Updated Review of Their Natural Sources, Synthesis, and Potentials. Antioxidants.

[12] Ou K., Gu L. (2014). Absorption and Metabolism of Proanthocyanidins. J. Funct. Foods.

[13] Zhang L., Wang Y., Li D., Ho C.-T., Li J., Wan X. (2016). The Absorption, Distribution, Metabolism and Excretion of Procyanidins. Food Funct..

[14] Rauf A., Imran M., Abu-Izneid T., Iahtisham-Ul-Haq, Patel S., Pan X., Naz S., Sanches Silva A., Saeed F., Rasul Suleria H.A. (2019). Proanthocyanidins: A Comprehensive Review. Biomed. Pharmacother..

[15] Patanè G.T., Putaggio S., Tellone E., Barreca D., Ficarra S., Maffei C., Calderaro A., Laganà G. (2023). Catechins and Proanthocyanidins Involvement in Metabolic Syndrome. Int. J. Mol. Sci..

[16] Nandakumar V., Singh T., Katiyar S.K. (2008). Multi-Targeted Prevention and Therapy of Cancer by Proanthocyanidins. Cancer Lett..

[17] Rasmussen S.E., Frederiksen H., Struntze Krogholm K., Poulsen L. (2005). Dietary Proanthocyanidins: Occurrence, Dietary Intake, Bioavailability, and Protection against Cardiovascular Disease. Mol. Nutr. Food Res..

[18] De La Iglesia R., Milagro F.I., Campión J., Boqué N., Martínez J.A. (2010). Healthy Properties of Proanthocyanidins. BioFactors.

[19] Janceva S., Andersone A., Lauberte L., Telysheva G., Krasilnikova J., Nokalne I., Janceva J. (2021). Influence of Extracts from Bark of Deciduous Trees on the Activity of the Amylolytic Enzyme—Alpha Amylase. Key Eng. Mater..

[20] Cires M.J., Wong X., Carrasco-Pozo C., Gotteland M. (2017). The Gastrointestinal Tract as a Key Target Organ for the Health-Promoting Effects of Dietary Proanthocyanidins. Front. Nutr..

[21] Dixon R.A., Xie D., Sharma S.B. (2005). Proanthocyanidins—A Final Frontier in Flavonoid Research?. New Phytol..

[22] Janceva S., Lauberte L., Arshanitsa A., Akishin J., Dizhbite T., Telysheva G. (2018). Optimization of Proanthocyanidins Extraction from Bark of Local Hardwood. Key Eng. Mater..

[23] Hellström J.K., Törrönen A.R., Mattila P.H. (2009). Proanthocyanidins in Common Food Products of Plant Origin. J. Agric. Food Chem..

[24] Smeriglio A., Barreca D., Bellocco E., Trombetta D. (2017). Proanthocyanidins and Hydrolysable Tannins: Occurrence, Dietary Intake and Pharmacological Effects. Br. J. Pharmacol..

[25] Platonova E.Y., Shaposhnikov M.V., Lee H.-Y., Lee J.-H., Min K.-J., Moskalev A. (2021). Black Chokeberry (*Aronia melanocarpa*) Extracts in Terms of Geroprotector Criteria. Trends Food Sci. Technol..

[26] Platibas-Aug-Lielakie-Smiltserksku-Aroniju-Un-Cidoniju-Lauki. https://lasi.lv/saimnieks-uznemejs/darzkopiba/platibas-aug-lielakie-smiltserksku-aroniju-un-cidoniju-lauki.4417.

[27] Janceva S., Andersone A., Lauberte L., Bikovens O., Nikolajeva V., Jashina L., Zaharova N., Telysheva G., Senkovs M., Rieksts G. (2022). Sea Buckthorn (*Hippophae rhamnoides*) Waste Biomass after Harvesting as a Source of Valuable Biologically Active Compounds with Nutraceutical and Antibacterial Potential. Plants.

[28] Andersone A., Janceva S., Lauberte L., Skadins I., Nikolajeva V., Logviss K., Zaharova N., Rieksts G., Telysheva G. (2023). A Comparative Analysis of the Proanthocyanidins from Fruit and Non-Fruit Trees and Shrubs of Northern Europe: Chemical Characteristics and Biological Activity. Sustain. Chem. Pharm..

[29] Milella R.A., De Rosso M., Gasparro M., Gigante I., Debiase G., Forleo L.R., Marsico A.D., Perniola R., Tutino V., Notarnicola M. (2023). Correlation between Antioxidant and Anticancer Activity and Phenolic Profile of New Apulian Table Grape Genotypes (*V. vinifera* L.). Front. Plant Sci..

[30] Cai Y., Luo Q., Sun M., Corke H. (2004). Antioxidant Activity and Phenolic Compounds of 112 Traditional Chinese Medicinal Plants Associated with Anticancer. Life Sci..

[31] González-Quilen C., Rodríguez-Gallego E., Beltrán-Debón R., Pinent M., Ardévol A., Blay M.T., Terra X. (2020). Health-Promoting Properties of Proanthocyanidins for Intestinal Dysfunction. Nutrients.

[32] Tamo A.K. (2024). Nanocellulose-Based Hydrogels as Versatile Materials with Interesting Functional Properties for Tissue Engineering Applications. J. Mater. Chem. B.

[33] Ioelovich M. (2021). Adjustment of Hydrophobic Properties of Cellulose Materials. Polymers.

[34] Rodríguez-Fabià S., Torstensen J., Johansson L., Syverud K. (2022). Hydrophobization of Lignocellulosic Materials Part II: Chemical Modification. Cellulose.

[35] Kabir S.M.F., Sikdar P.P., Haque B., Bhuiyan M.A.R., Ali A., Islam M.N. (2018). Cellulose-Based Hydrogel Materials: Chemistry, Properties and Their Prospective Applications. Prog. Biomater..

[36] Persano F., Malitesta C., Mazzotta E. (2024). Cellulose-Based Hydrogels for Wastewater Treatment: A Focus on Metal Ions Removal. Polymers.

[37] Mergbi M., Galloni M.G., Aboagye D., Elimian E., Su P., Ikram B.M., Nabgan W., Bedia J., Amor H.B., Contreras S. (2023). Valorization of Lignocellulosic Biomass into Sustainable Materials for Adsorption and Photocatalytic Applications in Water and Air Remediation. Environ. Sci. Pollut. Res..

[38] Patroklou G., Triantafyllopoulou E., Goula P.-E., Karali V., Chountoulesi M., Valsami G., Pispas S., Pippa N. (2025). pH-Responsive Hydrogels: Recent Advances in Pharmaceutical Applications. Polymers.

[39] Musialik M., Kuzmicz R., Pawłowski T.S., Litwinienko G. (2009). Acidity of Hydroxyl Groups: An Overlooked Influence on Antiradical Properties of Flavonoids. J. Org. Chem..

[40] Karagoz P., Khiawjan S., Marques M.P.C., Santzouk S., Bugg T.D.H., Lye G.J. (2024). Pharmaceutical Applications of Lignin-Derived Chemicals and Lignin-Based Materials: Linking Lignin Source and Processing with Clinical Indication. Biomass Conv. Bioref..

[41] Li Y., Ding X., Hu H., Xu F.-J. (2024). Stimulus-Responsive Polysaccharide-Based Hydrogels: From Design to Biomedical Applications. Precis. Med. Eng..

[42] Mahmoudi C., Tahraoui Douma N., Mahmoudi H., Iurciuc C.E., Popa M. (2024). Hydrogels Based on Proteins Cross-Linked with Carbonyl Derivatives of Polysaccharides, with Biomedical Applications. Int. J. Mol. Sci..

[43] De Oliveira I., Santos-Buelga C., Aquino Y., Barros L., Heleno S.A. (2025). New Frontiers in the Exploration of Phenolic Compounds and Other Bioactives as Natural Preservatives. Food Biosci..

[44] Hu M. (2016). Oxidative Stability and Shelf Life of Low-Moisture Foods. Oxidative Stability and Shelf Life of Foods Containing Oils and Fats.

[45] Li C., Wang Y., Yu W. (2011). Dynamic Changes of Phenolic Compound Contents in Leaf and Bark of Poplar during Autumn Temperature Drop. J. For. Res..

[46] Symma N., Hensel A. (2022). Advanced Analysis of Oligomeric Proanthocyanidins: Latest Approaches in Liquid Chromatography and Mass Spectrometry Based Analysis. Phytochem. Rev..

[47] Rockenbach I.I., Jungfer E., Ritter C., Santiago-Schübel B., Thiele B., Fett R., Galensa R. (2012). Characterization of Flavan-3-Ols in Seeds of Grape Pomace by CE, HPLC-DAD-MSn and LC-ESI-FTICR-MS. Food Res. Int..

[48] Renard C.M.G.C., Watrelot A.A., Le Bourvellec C. (2017). Interactions between Polyphenols and Polysaccharides: Mechanisms and Consequences in Food Processing and Digestion. Trends Food Sci. Technol..

[49] Choma J., Szczęśniak B., Jaroniec M. (2025). Mechanochemical Preparation of Biomass-Derived Porous Carbons. Molecules.

[50] Mungwari C.P., King’ondu C.K., Sigauke P., Obadele B.A. (2025). Conventional and Modern Techniques for Bioactive Compounds Recovery from Plants: Review. Sci. Afr..

[51] Comission Regulation (EU) 2023/915. https://eur-lex.europa.eu/legal-content/EN/TXT/PDF/?uri=CELEX:32023R0915.

[52] Janceva S., Svarta A., Nikolajeva V., Zaharova N., Rieksts G., Andersone A. (2025). Forest Logging Residue Valorization into Valuable Products According to Circular Bioeconomy. Forests.

[53] Zakis G. (1994). Conductometric Titration. Functional Analysis of Lignins and Their Derivatives.

[54] Porter L.J., Hrstich L.N., Chan B.G. (1985). The Conversion of Procyanidins and Prodelphinidins to Cyanidin and Delphinidin. Phytochemistry.

